# Clinical and Molecular Characterization of Hyperinsulinism in Kabuki Syndrome

**DOI:** 10.1210/jendso/bvae101

**Published:** 2024-05-21

**Authors:** Elizabeth Rosenfeld, Lauren M Mitteer, Kara Boodhansingh, Victoria R Sanders, Heather McKnight, Diva D De Leon

**Affiliations:** Congenital Hyperinsulinism Center, Division of Endocrinology and Diabetes, Children’s Hospital of Philadelphia, Philadelphia, PA 19104, USA; Department of Pediatrics, Perelman School of Medicine, University of Pennsylvania, Philadelphia, PA 19104, USA; Congenital Hyperinsulinism Center, Division of Endocrinology and Diabetes, Children’s Hospital of Philadelphia, Philadelphia, PA 19104, USA; Congenital Hyperinsulinism Center, Division of Endocrinology and Diabetes, Children’s Hospital of Philadelphia, Philadelphia, PA 19104, USA; Congenital Hyperinsulinism Center, Division of Endocrinology and Diabetes, Children’s Hospital of Philadelphia, Philadelphia, PA 19104, USA; Congenital Hyperinsulinism Center, Division of Endocrinology and Diabetes, Children’s Hospital of Philadelphia, Philadelphia, PA 19104, USA; Congenital Hyperinsulinism Center, Division of Endocrinology and Diabetes, Children’s Hospital of Philadelphia, Philadelphia, PA 19104, USA; Department of Pediatrics, Perelman School of Medicine, University of Pennsylvania, Philadelphia, PA 19104, USA

**Keywords:** *KMT2D*, *KDM6A*, hypoglycemia, insulin, diazoxide

## Abstract

**Context:**

Kabuki syndrome (KS) is associated with congenital hyperinsulinism (HI).

**Objective:**

To characterize the clinical and molecular features of HI in children with KS.

**Design:**

Retrospective cohort study of children with KS and HI evaluated between 1998 and 2023.

**Setting:**

The Congenital Hyperinsulinism Center of the Children's Hospital of Philadelphia.

**Patients:**

Thirty-three children with KS and HI.

**Main Outcome Measure(s):**

HI presentation, treatment, course, and genotype.

**Results:**

Hypoglycemia was recognized on the first day of life in 25 children (76%). Median age at HI diagnosis was 1.8 months (interquartile range [IQR], 0.6-6.1 months). Median age at KS diagnosis was 5 months (IQR, 2-14 months). Diagnosis of HI preceded KS diagnosis in 20 children (61%). Twenty-four children (73%) had a pathogenic variant in *KMT2D*, 5 children (15%) had a pathogenic variant in *KDM6A*, and 4 children (12%) had a clinical diagnosis of KS. Diazoxide trial was conducted in 25 children, 92% of whom were responsive. HI treatment was discontinued in 46% of the cohort at median age 2.8 years (IQR, 1.3-5.7 years).

**Conclusion:**

Hypoglycemia was recognized at birth in most children with KS and HI, but HI diagnosis was often delayed. HI was effectively managed with diazoxide in most children. In contrast to prior reports, the frequency of variants in *KMT2D* and *KDM6A* were similar to their overall prevalence in individuals with KS. Children diagnosed with KS should undergo evaluation for HI, and, because KS features may not be recognized in infancy, *KMT2D* and *KDM6A* should be included in the genetic evaluation of HI.

Kabuki syndrome (KS) is a congenital multisystem disorder with estimated prevalence of 1:32 000 live births [1]. Cardinal manifestations include characteristic facial features, skeletal anomalies, persistence of fetal fingertip pads, postnatal growth deficiency, and mild-to-moderate intellectual disability. Since the first descriptions of the syndrome by Niikawa et al and Kuroki et al in 1981 [2, 3], a wide and heterogenous phenotypic spectrum has been recognized. Additional common features include congenital heart defects, genitourinary and gastrointestinal anomalies, hypotonia, feeding difficulties, hearing loss, immune dysfunction, and endocrinopathy [4]. Among the endocrine manifestations of KS, congenital hyperinsulinism (HI) has increasingly been reported.

Neonatal/early infantile hypoglycemia has been described in 7% to 10% of children with KS [5, 6] and has been reported to persist into early childhood in 20% of reported cases [6]. In addition to HI, potential etiologies for neonatal hypoglycemia in KS include GH and/or cortisol deficiency [5, 7, 8]. The incidence of HI among children with KS has been estimated at 0.3% to 4% [4, 6]. Because these estimates are drawn from large case series in which details regarding evaluation and course are often not reported, the true incidence of HI in KS may be much higher. The mechanisms underlying hyperinsulinism in KS remain incompletely understood. Disruption of epigenetic changes during pancreatic differentiation and abnormal regulation of insulin secretion have been hypothesized [9, 10].

KS is caused by monoallelic loss of function variants in *KMT2D* or *KDM6A* [11-13]. Both genes encode histone-modifying proteins that interact with each other as part of the ASCOM complex to regulate gene expression [14]. *KMT2D* encodes a histone 3 lysine 4 methyltransferase, responsible for depositing activating methylation marks on chromatin, and *KDM6A* encodes a H3K27 demethylase, responsible for removing repressive epigenetic marks [15, 16]. Most individuals (>80%) with a molecular diagnosis of KS have heterozygous pathogenic variants in *KMT2D* (previously known as *MLL2*, autosomal dominant KS type 1, OMIM 147920), whereas 5% to 11% have heterozygous or hemizygous variants in *KDM6A* (previously known as *UTX*, X-linked dominant KS type 2, OMIM 300867) [4, 11, 17, 18]. Up to 30% of individuals with a clinical diagnosis of KS do not have a detectable variant in either gene [4]. HI has been reported to occur more frequently in children with *KDM6A* KS than *KMT2D* KS [18-20]. To date, the presentation of HI in children with KS has been described in a growing number of case series as well as a recent meta-analysis of previously published cases by Hoermann et al [19]. In this study, we aimed to characterize the clinical and molecular features of HI in a large single-center cohort of children with KS and HI.

## Methods

A retrospective chart review of children with KS and HI evaluated by the Congenital Hyperinsulinism Center of the Children's Hospital of Philadelphia (CHOP) between 1998 and 2023 was conducted. Demographic, clinical, biochemical, and molecular data were extracted from the electronic medical record. Diagnosis of KS was based on international consensus diagnostic criteria of history of infantile hypotonia, developmental delay and/or intellectual disability, and at least 1 of the following: (1) pathogenic or likely pathogenic variant in *KMT2D* or *KDM6A* and/or (2) typical dysmorphic features (long palpebral fissures, eversion of the lateral third of the lower eyelid, arched and broad eyebrows with lateral sparsening, short columella with depressed nasal tip, prominent ears, persistent fingertip pads) [4]. Diagnosis of HI was based on biochemical evidence of inappropriate insulin action at the time of hypoglycemia (plasma glucose <50 mg/dL [2.8 mmol/L]), as previously described [21]. Diazoxide is the first-line treatment for HI [22]. Given the risk of diazoxide-associated fluid retention and pulmonary hypertension, our Center's practice is to concomitantly treat with diuretic and consult cardiology before initiating diazoxide in children with congenital heart disease. Diazoxide responsiveness was defined as the ability to maintain plasma glucose concentration >70 mg/dL (3.9 mmol/L) for at least 12 hours of fasting and/or generate appropriate hyperketonemia (plasma β-hydroxybutyrate >1.8 mmol/L) before development of plasma glucose <50-60 mg/dL (2.8-3.3 mmol/L) [22, 23]. Resolution of HI was defined as demonstration of the development of hyperketonemia (β-hydroxybutyrate >1.8 mmol/L) before development of plasma glucose <50 mg/dL (2.8 mmol/L) during a controlled inpatient fast performed off treatment [22]. Age at HI diagnosis was calculated from the date of birth and date of “critical sample” laboratory specimen. Age at last follow-up was calculated from the date of birth and last contact date.

### Molecular Analysis

Molecular analysis for the genes associated with KS, *KMT2D* and *KDM6A*, was performed on genomic DNA in commercial laboratories for all children in the cohort except in 2 (patients 1 and 29) who were screened on a research basis. For the research sequencing, barcoded libraries were prepared from 80 ng of gDNA using the Ion AmpliSeq Library Kit 2.0 (Thermo Fisher cat. no. 4480442) and Ion AmpliSeq Comprehensive Cancer Panel (cat. no. 4477685) and sequenced on an Ion Torrent S5 sequencing system using the Ion 540 Kit-OT2 and Ion 540 Chip (A27753 and A27766). Aligned reads in BAM format were uploaded to the cloud-based Ion Reporter Software (Thermo Fisher, https://ionreporter.thermofisher.com/) for variant calling using the version 5.10 Comprehensive Cancer Panel germline and single sample somatic variant calling workflows.

As detailed in Table 1, variants in the KS genes, *KMT2D* and *KDM6A*, were identified by either Sanger sequencing of the KS genes (n = 1), targeted next-generation sequencing (NGS) of the KS genes (n = 14), targeted NGS of a HI gene panel (n = 2), clinical whole-exome sequencing (n = 10), or single nucleotide polymorphism array (n = 1). Type of mutation analysis was unknown for 3 children. Genetic testing for KS genes was negative in 2 children who were evaluated by Sanger sequencing and comparative genomic hybridization array (patient 4) and research NGS (patient 29). The functional consequences of novel, missense mutations were predicted with bioinformatics software SIFT [32] and PolyPhen2 [33] and searched against the gnomAD Browser (v4.0) [34].

**Table 1.** *KDM6A* and *KMT2D* variants identified in 31 children with Kabuki syndrome and hyperinsulinism

Patient	Type of gene screening	Variant	Variant type	Allele	Zygosity	Reference
1	NGS (KS genes)	*KDM6A* c.1085_1086delAT/p.Tyr362*	Termination	n.d.	Het	—
19	NGS (HI panel)	*KDM6A* c.2982del/p.Asn994Lysfs*5	Frameshift	n.d.	Het	—
7	WES	*KDM6A* c.3016C > T/p.Gln1006*	Termination	Non-maternal*^a^*	Het	—
9	NGS (KS genes)	*KDM6A* c.3717G > A/p.Trp1239*	Termination	n.d.	Het	[24, 25]
24	SNP array	0.5 Mb deletion of proximal Xp11.3 including *KDM6A*	Deletion	n.d.		—
14	NGS (KS genes)	*KMT2D* c.349C > T/p.Gln117*	Termination	n.d.	Het	—
25	WES	*KMT2D* c.970dupC/p.Arg324Profs*18	Frameshift	de novo	Het	—
22	WES	*KMT2D* c.1394C > T/p.Ser465*	Termination	de novo	Het	—
16	NGS (KS genes)	*KMT2D* c.1468_1471del/p.Glu490Asnfs*439	Frameshift	n.d.	Het	—
26	WES	*KMT2D* c.3754C > T/p.Arg1252*	Termination	n.d.	Het	[26, 27]
28	NGS (KS genes)	*KMT2D* c.4843C > T/p.Arg1615*	Termination	n.d.	Het	[12]
11	NGS (KS genes)	*KMT2D* c.5104C > T/p.Arg1702*	Termination	n.d.	Het	—
6	NGS (KS genes)	*KMT2D* c.5135delA/p.Lys1712Argfs*10	Frameshift	n.d.	Het	—
12	NGS (KS genes)	*KMT2D* c.6313C > T/p.Arg2105Cys*^b^*	Missense	Maternal	Het	
33	WES	*KMT2D* c.6595del/p.Tyr2199Ilefs*65	Frameshift	De novo	Het	[28]
17	NGS (HI panel)	*KMT2D* c.6844C > T/p.Arg2282Trp*^c^*	Missense	n.d.	Het	—
8	NGS (KS genes)	*KMT2D* c.7479delG/p.Phe2494fs	Frameshift	n.d.	Het	[29]
18	n.d.	*KMT2D* c.9773delA/p.Lys3258fs*72	Frameshift	n.d.	Het	—
23	WES	*KMT2D* c.9947delG/p.Gly3316Valfs*14	Frameshift	De novo	Het	—
10	NGS (KS genes)	*KMT2D* c.10201C > T/p.Gln3401*	Termination	n.d.	Het	—
2	Sanger (KS genes)	*KMT2D* c.10999C > T/p.Gln3667*	Termination	n.d.	Het	—
15	NGS (KS genes)	*KMT2D* c.11944C > T/p.Arg3982*	Termination	n.d.	Het	[24, 30]
21	n.d.	*KMT2D* c.12592C > T/p.Arg4196*	Termination	De novo	Het	[29, 31]
3	NGS (KS genes)	*KMT2D* c.12835_12848del/p.Ala4279Serfs*50	Frameshift	n.d.	Het	—
31	WES	*KMT2D* c.12862del/p.Arg4288Glyfs*96	Frameshift	De novo	Het	—
20	n.d.	*KMT2D* c.12878_12893del/p.Pro4293fs	Frameshift	n.d.		—
30	WES	*KMT2D* c.13606C > T/p.Arg4536*	Termination	De novo	Het	[12]
27	WES	*KMT2D* c.13780del/p.Ala4594Profs*23	Frameshift	De novo		—
5	NGS (KS genes)	*KMT2D* c.15088C > T/p.Arg5030Cys	Missense	n.d.	Het	[24, 29]
13	NGS (KS genes)	*KMT2D* c.16342C > T/p.Arg5448*	Termination	De novo		[28]
32	WES	*KMT2D* c.16416_16417delCA/p.Ile5473*	Termination	De novo	Het	—

The nucleotides of *KMT2D* and corresponding amino acids were numbered according to the sequence RefSeq NM_003482.3 according to HGVS. The nucleotides of *KDM6A* and corresponding amino acids were numbered according to NM_021140.3 according to HGVS.

Abbreviations: Het, heterozygous; HI, hyperinsulinism; KS, Kawasaki syndrome; n.d., data not available; NGS, next-generation sequencing; SNP, single nucleotide polymorphism; WES, whole-exome sequencing.

^*a*^Paternal DNA not available for testing.

^*b*^Classified as likely benign.

^*c*^Classified as a variant of uncertain significance.

Molecular analysis for nonsyndromic genes associated with HI was performed in commercial laboratories in 24 children. Of these, 3 children had Sanger sequencing of *ABCC8*, *KCNJ11*, *GCK,* and *GLUD1* (patients 2, 5, and 29), 11 children had targeted NGS panels that included *ABCC8*, *KCNJ11*, *GCK*, *GLUD1*, *HNF1A*, *HNF4A*, *HADH*, *SLC16A1*, and *UCP2* (patients 3, 6, 8-10, 12, 14-15, 17, 19, and 26), and 10 children had whole-exome sequencing (patients 4, 7, 22-23, 25, 27, and 30-33).

### Statistical Analysis

Cohort characteristics were summarized using descriptive statistics, including counts and percentages for categorical variables, means and standard deviations (SDs) for normally distributed continuous variables, and median and interquartile range (IQR) for nonnormally distributed continuous variables. Wilcoxon rank-sum test was used to compare age at last follow-up between children who discontinued HI treatment and those who had not, as well as to compare clinical features of HI between children with variants in *KMT2D* and *KDM6A*. One-sample, 2-sided *z*-test of proportions was used to compare the observed proportion of children with HI and KS resulting from *KDM6A* variants to the expected frequency of *KDM6A* variants among children with molecularly confirmed KS. An expected frequency of 11% was chosen for comparison because this is the frequency of *KDM6A* variants among children with molecularly confirmed KS reported in the Kabuki Syndrome: International Consensus Diagnostic Criteria [4], the most comprehensive systematic review of molecularly confirmed KS to date. A *P* value <.05 was set as the threshold for statistical significance. Statistical analyses were performed using Stata 15.1 (Stata Corp., College Station, TX).

The study was reviewed and approved by the CHOP Institutional Review Board.

## Results

A total of 33 children (19 females, 14 males) with both KS and HI were evaluated by the CHOP Congenital Hyperinsulinism Center between 1998 and 2023. Race was documented as Asian in 9% of the cohort, White in 55%, more than 1 race in 12%, and was unknown/undocumented in 24% of the cohort. Ethnicity was documented as Hispanic or Latino in 6% of the cohort, not Hispanic or Latino in 76%, and was unknown/undocumented in 18% of the cohort. Most children were born at term (median gestational age, 37 weeks [IQR, 35-38 weeks]) with appropriate for gestational age birth weight (median birth weight, 3.1 kg [IQR, 2.5-3.5 kg]). Additional clinical characteristics of the cohort are summarized in Table 2. Median age at last follow-up was 2.6 years and ranged from 4 months to 14 years. Hypoglycemia was recognized on the first day of life in 25 children (76%). Among the 8 children for whom hypoglycemia was recognized after the first day of life, median age of presentation was 2.6 months (IQR, 0.5-5.1 months); hypoglycemia was identified incidentally on outpatient laboratory results in 3 children, during enteral tube feed wean in 2 children, during admission for viral illness in 1 child, and in 2 children, features of the initial presentation were not documented. Median age of HI diagnosis in the overall cohort was 1.8 months (IQR, 0.6-6.1 months). In 6 children, HI was not diagnosed until after age 1 year. Median time from first recorded hypoglycemia episode to HI diagnosis was 1.0 months (IQR, 5 days-3.9 months; range, 0-28 months).

**Table 2.** Clinical characteristics of cohort

Patient	Sex	KS Gene	BW	Age KS dx (mo)	Age HI dx (mo)	DZ resp	HI management	Age Follow-up (Y)	Current status
1	M	*KDM6A*	LGA	24	2	n.d.	n.d.	6.9	HI reported as resolved by age 6 y. Fast data not available.
2	M	*KMT2D*	AGA	4	28.3	Y	Diazoxide 15 mg/kg/d	14.0	HI resolved at age 10 y, 6 mo
3	M	*KMT2D*	AGA	60	3	Y	Diazoxide 5 mg/kg/d	9.5	Diazoxide 5 mg/kg/d
4	M	Clinical	AGA	7	1.1	N	Intragastric dextrose GIR 4 mg/kg/min	1.0	HI resolved at age 8.5 mo
5	M	*KMT2D*	AGA	5	1	Y	Fluid overload on diazoxide; intragastric dextrose GIR 4.4 mg/kg/min	8.5	Treatment discontinued at age 12 mo. Evaluation not performed, home fasting glucoses >70 mg/dL.
6	M	*KMT2D*	LGA	5	0.5	Y	Diazoxide 15 mg/kg/d	9.2	Treatment discontinued at age 9 years. Steroid-induced hyperglycemia.
7	F	*KDM6A*	AGA	59	0.3	Y	Diazoxide 10 mg/kg/d	7.6	HI resolved at age 5 y
8	F	*KMT2D*	AGA	14	22	Y	Diazoxide 7.5 mg/kg/d	7.4	Diazoxide 6.9 mg/kg/d
9	F	*KDM6A*	AGA	20	20	n/a	GT feeds	7.2	HI resolved at age 5 y, 8 mo
10	F	*KMT2D*	AGA	5	3.9	N	Intragastric dextrose GIR 6.5 mg/kg/min; lanreotide started at age 1 y	7.0	HI resolved at age 6 y, 6 mo
11	F	*KMT2D*	AGA	14	14.4	n/a	GT feeds	4.7	HI not requiring treatment at age 2 y, 5 mo (fasted 11 hours with glucose > 70 mg/dL)
12	M	Clinical	LGA	1	0.6	Y	Diazoxide 5 mg/kg/d	2.6	Diazoxide 5 mg/kg/d
13	M	*KMT2D*	AGA	5	0.2	Y	Diazoxide 4.7 mg/kg/d	1.3	Treatment discontinued at age 15 mo Evaluation not performed, home fasting glucoses >70 mg/dL
14	F	*KMT2D*	AGA	4	3.9	Y	Diazoxide 18 mg/kg/d	0.7	Diazoxide 16.9 mg/kg/d
15	M	*KMT2D*	AGA	5	0.1	n/a*^a^*	Intragastric dextrose GIR 3.9 mg/kg/min	4.6	HI not requiring treatment at age 19 mo (fasting 13 hours with glucose > 70 mg/dL)
16	M	*KMT2D*	n.d.	Before HI dx	19	Y	Diazoxide 15 mg/kg/d	3.6	HI resolved at age 3 y, 2 mo
17	F	Clinical	n.d.	3	1.9	Y	Diazoxide (dose unknown)	0.4	Diazoxide (dose unknown)
18	F	*KMT2D*	n.d.	4	4	n/a*^a^*	GT feeds	0.4	GT feeds
19	F	*KDM6A*	AGA	2	2	n/a*^a^*	Intragastric dextrose GIR 2 mg/kg/min + continuous feeds because of intolerance	3.5	HI resolved at age 3 y, 2 mo
20	F	*KMT2D*	AGA	7	6	n/a*^a^*	GT feeds	4.3	GT feeds
21	F	*KMT2D*	LGA	18	16	Y	Cytopenia on diazoxide; lanreotide 60 mg monthly	4.0	Lanreotide 60 mg monthly
22	M	*KMT2D*	AGA	4	0.5	Y	Diazoxide 8.7 mg/kg/d	3.1	Diazoxide 6.4 mg/kg/d
23	F	*KMT2D*	AGA	1	0.8	Y	Diazoxide 7.5 mg/kg/d	2.3	Diazoxide 10.6 mg/kg/d
24	F	*Xp11.3 del incl KDM6A*	AGA	1	1	Y	Diazoxide 14 mg/kg/d	2.0	Treatment discontinued at age 23 mo. Evaluation not performed, home fasting glucoses >70 mg/dL
25	F	*KMT2D*	AGA	0.5	0.2	Y	Diazoxide 9.5 mg/kg/d	2.3	Diazoxide 15 mg/kg/d
26	F	*KMT2D*	AGA	1	0.3	Y	Diazoxide 7 mg/kg/d	2.1	Diazoxide 9.75 mg/kg/d
27	M	*KMT2D*	AGA	6	6.2	Y	Diazoxide 7.8 mg/kg/d	1.5	Diazoxide 6.45 mg/kg/d
28	M	*KMT2D*	AGA	0.75	0.8	Y	Diazoxide 7 mg/kg/d	1.5	HI resolved at age 8 mo
29	F	Clinical	AGA	21	21.9	Y	Diazoxide 12.5 mg/kg/d	2.0	Diazoxide 12.5 mg/kg/d
30	M	*KMT2D*	AGA	2	1.1	Y	Diazoxide 8 mg/kg/d	1.5	Diazoxide 4.9 mg/kg/d
31	F	*KMT2D*	SGA	7	6.1	Y	Diazoxide 10 mg/kg/d	1.8	Diazoxide 6.6 mg/kg/d
32	F	*KMT2D*	AGA	9	0.3	n/a	Intragastric dextrose overnight GIR 2 mg/kg/min	1.9	Intragastric dextrose overnight GIR 1.3 mg/kg/min
33	F	*KMT2D*	AGA	1	1.7	Y	Diazoxide 7.5 mg/kg/d	0.9	Diazoxide 11.6 mg/kg/d

Abbreviations: AGA, appropriate for gestational age; BW, birth weight; dx, diagnosis; DZ, diazoxide; GIR, glucose infusion rate; GT, gastrostomy tube; HI, hyperinsulinism; KS, Kabuki syndrome; LGA, large for gestational age; n/a, not applicable; n.d., data not available; SGA, small for gestational age.

^*a*^Trial not conducted because of congenital heart defect.

### HI Treatment and Course

Of the 25 children in whom a trial of diazoxide was conducted, 23 (92%) were diazoxide responsive. Median initial diazoxide dose was 8.4 mg/kg/day (IQR, 7.3-13.3 mg/kg/day). The 2 children with diazoxide-unresponsive HI were treated with continuous intragastric dextrose. Diazoxide trial was not conducted because of cardiac comorbidity in 4 children (hypoplastic left heart syndrome, ventricular septal defect with aortic arch hypoplasia, and coarctation of aorta), feeding intolerance necessitating continuous enteral feeds on which hypoglycemia was adequately controlled in 2 children, and family preference to treat with continuous intragastric dextrose in 1 child. In 1 child, initial HI treatment was unknown. Among those responsive to diazoxide, treatment was discontinued in 2 children because of adverse effects: 1 child developed fluid overload, without pulmonary hypertension, despite aggressive diuresis and was treated with continuous intragastric dextrose, and the other developed persistent neutropenia and anemia and was transitioned to lanreotide 60 mg monthly at 16 months of age. Among the 6 children treated with continuous intragastric dextrose, median glucose infusion rate was 4.0 mg/kg/min (IQR, 2.0-4.4 mg/kg/min). Four children, all of whom required frequent and/or continuous gastrostomy tube feeds because of severe feeding intolerance, were managed with enteral feeds alone.

HI treatment was discontinued over the follow-up period in 15 children (46%) at median age 2.8 years (IQR, 1.3-5.7 years; range, 0.6-10.5 years). Of the children who discontinued treatment, biochemical resolution of HI was demonstrated on inpatient fasting evaluation in 8 children (median age resolution, 4.1 years [IQR, 1.9-6.1 years]), and normal plasma glucose (>70 mg/dL [3.9 mmol/L]) after an overnight fast was reported in the remainder. Children off HI treatment were older at time of last follow-up than those still on treatment (median age, 4.7 years [IQR, 2.0-7.6 years] vs 2.0 years [IQR, 1.5-3.1 years], *P* = .02, Wilcoxon rank-sum test).

Molecular analysis for genes associated with nonsyndromic forms of HI revealed a maternally inherited *KCNJ11* variant in 1 child (c.868G > A/p.Val290Met, patient 5). There was no reported history of HI in his mother. This variant has been previously reported in the heterozygous state in children with focal HI and in the homozygous state in a child with diffuse HI [35, 36], suggesting that it acts recessively. Based on these findings, this variant was considered unlikely to explain the HI phenotype in this child.

### KS Genotype and Clinical Features

Diagnosis of KS was established at median age 5 months (IQR, 2-14 months). HI diagnosis was established before the diagnosis of KS in 61% of the cohort. As shown in Table 2, 24 children had a pathogenic variant identified in *KMT2D* (73%; 13 females, 11 males) and 5 children (15%; 4 females, 1 male) had a pathogenic variant identified in *KDM6A*. The majority of pathogenic variants identified in *KMT2D* and *KDM6A* resulted in an early termination codon and truncated protein (n = 27). Of the remaining pathogenic variants identified, 1 child (patient 24) had a 0.5-Mb chromosomal deletion involving proximal Xp11.3, including the *KDM6A* locus, and 1 child (patient 5) had a missense variant in *KMT2D* (c.15088C > T/p.Arg5030Cys) previously reported to be disease causing [24, 29]. The remaining 4 children had a clinical diagnosis of KS (12%; 2 females, 2 males). Of the children with a clinical diagnosis, 1 (patient 12) had a missense variant identified in *KMT2D* classified as likely benign, 1 (patient 17) had a missense variant of uncertain significance identified in *KMT2D*, and 2 (patients 4 and 29) had negative screening for variants in *KMT2D* and *KDM6A*. In patient 12, the missense variant identified in *KMT2D* (c.6313C > T/p.Arg2105Cys) was inherited from his apparently unaffected mother and was thus classified as likely benign. The missense variant of uncertain significance in *KMT2D* (c.6844C >T/p.Arg2282Trp) identified in patient 17 has not been reported to be associated with KS, is predicted to be pathogenic by PolyPhen [33], and is present in control populations at a maximum population frequency of 1:1980 in the South Asian subpopulation in gnomAD v4.0 [34].

Typical facial features were documented over the follow-up period in 91% of children: long palpebral fissures in 67%, eversion of the lateral third of the lower eyelid in 21%, arched and broad eyebrows in 45%, short columella with depressed nasal tip in 15%, prominent ears in 61% of children. Persistent fingertip pads were documented in 61% of children. Feeding difficulties and infantile hypotonia were common, present in 88% and 79% of children, respectively. Congenital heart defects were present in 64%, ophthalmologic abnormalities in 48%, genitourinary abnormalities in 45%, microcephaly in 36%, congenital hypothyroidism in 21%, immunologic abnormalities in 15%, and seizures in 15% of children. Postnatal growth restriction/short stature was identified over the follow-up period in 61% of children.

### Comparison of HI Clinical Features Between Children with *KMT2D* and *KDM6A* KS

In our cohort, 17% of children with molecularly confirmed KS had pathogenic variants in *KDM6A* compared to expected frequency of 11% (17% vs 11%, *P* = .31, 1-sample, 2-sided *z*-test of proportions). Median age of HI presentation did not differ between children with *KDM6A* and *KMT2D* KS (1 day [IQR, 1-1 day] vs 1 day [IQR, 1-8 days], *P* = .18, Wilcoxon rank-sum test). However, all 8 children in whom hypoglycemia was recognized after the first day of life had variants in *KMT2D*. Differences in median age at HI diagnosis were not observed between children with pathogenic variants in *KDM6A* and *KMT2D* (1.8 months [IQR, 1.0-2.0 months] vs 2.3 months [IQR, 0.4-6.2 months], *P* = .98, Wilcoxon rank-sum test). Of the 2 children with diazoxide-unresponsive HI, 1 harbored a *KMT2D* pathogenic variant and the other had a clinical diagnosis. HI treatment was discontinued over the follow-up period in 9 children (38%) with pathogenic variants in *KMT2D* and in all 5 children with pathogenic variants in *KDM6A.* Children with variants in *KDM6A* were numerically, although not statistically significantly, older at last follow-up than those with variants in *KMT2D* (6.9 years [IQR, 3.5-7.2 years] vs 2.7 years [IQR, 1.5-5.9 years], *P* = .27, Wilcoxon rank-sum test).

## Discussion

We describe the clinical and molecular characteristics of 33 children with HI and KS evaluated at a single center. We found that hypoglycemia was identified on the first day of life in more than three-quarters of the cohort. These findings are in keeping with existing reports on the presentation of HI in KS. In a meta-analysis of 24 children with HI and KS, Hoermann et al found that 83% of children, for whom data were available, presented with hypoglycemia on the first day of life [19]. Despite early recognition of hypoglycemia in our cohort, diagnosis of HI was often delayed. Differentiating between the transitional neonatal hypoglycemia observed in healthy newborns and persistent hypoglycemia disorders—such as HI—on the first days of life poses a significant challenge in clinical practice and may have contributed to the delay in diagnosis observed within our cohort.

The prevalence of HI in children with KS was initially estimated to be 0.3% in a 2004 study by Genevieve et al, in which 1 child was diagnosed with HI of 313 published cases [6]. Since then, HI in children with KS has increasingly been described in case reports and series, yielding a more recent prevalence estimate of 4% in a large cohort of 449 children with molecularly confirmed KS [4]. However, these estimates reflect an assumption that individuals who did not undergo evaluation for HI, or for whom data were not reported, did not have the outcome. True absence of HI in these cases is thus uncertain, especially because signs and symptoms of hypoglycemia in infants are nonspecific, may be unrecognized, and are often incompletely evaluated. Taken together with findings in our cohort of delayed diagnosis of HI, these data suggest that HI may be underdiagnosed in children with KS. Additionally, because diagnosis of KS is often delayed beyond the first few months of life, as observed in our cohort and others [19, 20], it is plausible that mild cases of HI may remain undetected and resolve before the KS diagnosis is established, contributing to an underestimation of the true prevalence of HI among children with KS.

An increased likelihood of HI has been described in children with KS caused by pathogenic variants in *KDM6A* compared to those with pathogenic variants in *KMT2D* [17-20]. Yap et al found that of 11 children with HI and KS, 5 (46%) had variants in *KDM6A* [20]. Adam et al and Hoermann et al reported similar distributions—39% and 50% of children with HI and molecularly confirmed KS harbored variants in *KDM6A*, respectively [4, 19]. In a recent series of 80 individuals with *KDM6A* KS, HI was reported in 28% [37]. Compared to the general frequency of *KDM6A* variants in individuals with KS of 5% to 11%, *KDM6A* variants were significantly overrepresented among children with HI in these studies [4, 17].

In contrast, we found that frequency of variants in *KMT2D* (83%) and *KDM6A* (17%) in children with molecularly confirmed KS and HI did not significantly differ from the general prevalence of these variants in KS. Possible reasons for the observed difference include referral bias and differential ascertainment of the HI outcome between children included in our study—conducted at a large HI referral center—and cases assessed via systematic review of published reports, in which the completeness and accuracy of outcome data are less certain. It has also been suggested that documented cases of HI in KS represent individuals with more severe and persistent HI, whereas those with milder forms of HI remain unreported and/or undetected. Significant differences in HI clinical features were not observed between children with variants in *KMT2D* and *KDM6A* in our study nor in that of Hoermann et al [19]. However, in both studies, all children whose hypoglycemia was recognized after the first day of life harbored variants in *KMT2D*, potentially suggesting that differences in severity—and thus detection—may be present.

Aligned with existing literature, most children in our cohort were adequately treated with diazoxide. Ninety-two percent of children were diazoxide responsive, and treatment was generally well-tolerated. Despite a high-frequency of comorbidities, including congenital heart defects in 64%, the frequency of adverse effects prompting diazoxide discontinuation in our cohort—edema in 1/33 (3%) and cytopenia in 1/33 (3%)—paralleled those observed in children with HI because of other etiologies [38, 39]. Notably, a diuretic (typically chlorothiazide) is started concomitantly with diazoxide in all children treated at our center. Additionally, cardiology consultation is performed for children with cardiac comorbidity, and diazoxide trial was not conducted in 4 children who had clinically significant left ventricular outflow tract malformations.

Within our cohort of children with HI and KS, the spectrum and frequency of non-HI phenotypic features, including facial features, postnatal growth and feeding difficulties, and cardiac, ophthalmologic, and genitourinary structural differences, aligned with those typically observed in KS [4, 40]. These findings are in keeping with existing literature [19, 20], supporting that children with HI and KS do not appear to have specific phenotypic features that differentiate them from children with KS but without HI.

It is well-appreciated that the clinical diagnosis of KS can be challenging in young infants because typical features, particularly characteristic facial features, may not be present, or may be difficult to recognize in neonates [41]. In our cohort, 61% of children were diagnosed with HI before a KS diagnosis was established. These findings underscore the importance of including KS in the differential diagnosis of HI and highlight the value of including KS-associated genes in the genetic evaluation of HI. Timely diagnosis of KS is of particular importance because KS is associated with comorbidities that require multidisciplinary evaluation, longitudinal surveillance, and management. Furthermore, children diagnosed with KS should be evaluated for hypoglycemia, and if present, undergo formal evaluation to determine the etiology. Prompt identification and treatment of hypoglycemia is critical to mitigate the adverse neurocognitive outcomes associated with HI [42, 43]. This is especially important for children with KS because developmental delay and intellectual disability are cardinal features of the syndrome. Indeed, delayed diagnosis of HI among children with KS, as observed in our cohort, could be an important contributor to the neurodevelopmental impairment seen in KS.

Strengths of this study include the large number of children with HI and KS, comprehensive evaluation, and detailed characterization of clinical features. Limitations include those inherent to retrospective chart review, including omissions in documentation in the electronic medical record. As discussed previously, this was a single-center study conducted at a large HI referral center and was thus subject to both referral and ascertainment bias. Differential ascertainment of the HI outcome among children included in our study, compared to those reported previously in the literature, is plausible given our institutional practice and expertise in the evaluation of hypoglycemia. Consequently, findings may not be generalizable to different populations.

In conclusion, HI frequently presents on the first day of life in children with KS. Despite this, diagnosis of both HI and KS in affected children is often delayed. Diazoxide is usually effective and well-tolerated. Although concomitant use of diuretic with diazoxide is recommended for all infants, this is particularly important in children with KS, given the high frequency of cardiac comorbidities in this population. In contrast to prior studies, overrepresentation of *KDM6A* variants were not observed in this cohort of children with HI and KS. Although this finding warrants further investigation, we recommend that all children diagnosed with KS—regardless of genotype—should be screened for HI. Last, because KS features may not be recognized in infancy, *KMT2D* and *KDM6A* should be included in the genetic evaluation of HI.

## Data Availability

Restrictions apply to the availability of some or all data generated or analyzed during this study to preserve patient confidentiality or because they were used under license. The corresponding author will on request detail the restrictions and any conditions under which access to some data may be provided.

## Abbreviations

CHOP: Children's Hospital of Philadelphia
HI: hyperinsulinism
IQR: interquartile range
KS: Kabuki syndrome
NGS: next-generation sequencing
